# Annotations

**Published:** 1897-12-11

**Authors:** 


### ANNOTATIONS.

### A Municipal Milk Supply.

The municipalisation of industries has long been the dream of social reformers. The Fabians and their allies have maintained with much energy and many smart arguments that all instruments of production and distribution should be in the hands of the People spelt with a big P, while the people, spelt with a little p, should do the work at municipal wages, and buy the products at municipal prices; and among the industries which have seemed to some peculiarly appropriate for such experiment has been that of dairy farming. All of which is by the way. We have not arrived at that yet, although the restrictions and control to which the milk trade seems likely to have to submit before we can feel assured of getting pure milk are so elaborate that we hardly wonder at people asking whether it would not be simpler to municipalise the whole affair at once. Still, if we have not yet quite reached the consummation hoped for by our friends the Fabians, a big stride in that direction seems to be imminent in Bristol. Bristol, like many other towns, has laid down excellent rules for the regulation of all dairies within its boundaries; but, like other towns, it has to draw its milk supply from far and wide, from places over which its officers have no control, and the recent epidemic has been a lesson to it. The proposal is therefore made for the Health Committee to use their influence to form a strong local committee, who should employ the necessary officers for the inspection of the farms and the control of the arrangements. An association would be formed, which it is hoped that all the best dairymen would join; lists would be kept of farms under the sanction of the committee, so that anyone could see whether he was supplied from a "controlled" farm, and farmers who refused to be controlled would be left to the boycott of public opinion. Of course, all this inspection and control of farms is successfully worked at the present time by many of the large dairy companies. The interest of the Bristol scheme lies in the fact that it is to originate from the Health Committee, and that it is hoped that this committee will allow some of its members to serve on the Milk Committee, so giving it a definite connection with the corporation. This is one step. Municipalisation is but another!

### Women's Work and Men's Wages.

One of the speakers at the National Council of Women's Trades said that where women compete with men the wages of the inferior workmen will fall, but the better workmen will have less competition, and therefore their wages will rise. Where the employment of cheap workers, which women usually are, tends to increase an industry as a whole, this may be the case; but many industries cannot expand indefinitely, and in any case the immediate effect of the employment of women in an industry which has hitherto been confined to men is to send an equal number of men to seek other kinds of work. We do not mean to say, as some do, that therefore women should not seek employment. But it is worth while to ask if all the women who are engaged in our factories and shops are either economically or morally justified in entering the ranks of wage-earners, for it is not quite certain that a woman or her family is always richer because she brings in money from the outside. There is very rarely any excuse for a married woman entering the ranks of wage-earners. If she goes out to work, she must more or less neglect her home. If one could analyse the expenses of a family where the wife goes out to work and one where she does not, it might come out that the former is not much, if at all, the richer of the two. If women must make money, as in some cases they must, it is better that they should enter some work where they do not compete with men. There has never been any outcry against nurses, just because the work which they do is one peculiarly suited to their sex, and in it they have really developed a new industry, which is a benefit to all, and robs none.

### Post-Graduate Teaching in London.

The question of post-graduate teaching in London is one which must sooner or later be dealt with on a large and comprehensive scale. What is wanted is organisation, concentration, and punctuality. Foreigners and other visitors to London have neither the time nor the inclination to wander all over the town to the different hospitals, to find, perhaps, that the particular man they want to hear is away, or that he is late, or that the subject on which he is about to lecture is not known. So far as operative surgery and clinical demonstrations of acute cases are concerned, some central bureau of information, where strangers could find out what is going on and obtain authorisation to attend the various clinics, is, perhaps, all that can be done. But when it becomes a question of special courses, arranged on purpose for post-graduates, we cannot but think that something much more than a mere bureau is required. The difficulty lies in the fact that some lecturers are good and some are bad; some would draw together a class from all corners of the earth; while to hear others no one would go across the street, and we fear that in but few of the hospitals would it be possible to arrange a course to join in which all the members of the staff were not invited, while in some, if they were all invited, the course would be ruined. The most hopeful project, although at present it can hardly be called a project, is the establishment in some central position of a post-graduate laboratory and demonstration hall, managed by a strong committee who should run it on economical principles, selecting the most attractive lecturers and giving those the go-by who ceased to draw. Unfortunately, such a scheme would, we fear, be thought by some to have about it a dash of the unprofessional; and, of course, if the leaders could not be induced to join it, the whole thing would be quite impossible. But if such a thing could be arranged on proper professional lines, it might quickly make London a great centre for post-graduate teaching. The desire, and we may add the necessity, for such post-graduate teaching is growing every year, and it seems a great pity that London, with its vast clinical field, should not make itself the centre to which people in search of this teaching should flock from all parts of the English-speaking world. As things are at present this is quite out of the question, and people drift away to other places.

